# Mechanistic rationales for targeting interleukin-17A in spondyloarthritis

**DOI:** 10.1186/s13075-017-1249-5

**Published:** 2017-03-08

**Authors:** Siba P. Raychaudhuri, Smriti K. Raychaudhuri

**Affiliations:** 1 VA Medical Center Sacramento, Division of Rheumatology & Immunology, Sacramento, CA USA; 2 Division of Rheumatology, Allergy & Clinical immunology, University of California School of Medicine, Davis, & VA Medical Center Sacramento, Sacramento, CA USA

**Keywords:** Spondyloarthritis, Review, Interleukin-17, IL-17A inhibition, Ankylosing spondylitis, Psoriatic arthritis, Preclinical studies, Clinical practice

## Abstract

The term spondyloarthritis (SpA) is used to describe a group of inflammatory autoimmune diseases, including ankylosing spondylitis and psoriatic arthritis, with common genetic risk factors and clinical features. SpA is clinically distinct from rheumatoid arthritis and typically affects the spine, sacroiliac joints, entheses, and, less commonly, peripheral joints. Although the pathogenesis of SpA is not fully understood, recent findings have identified the interleukin (IL)-17 pathway as a key mediator of disease pathogenesis. Clinical evidence for the efficacy of IL-17A inhibition by biologic agents was initially shown in patients with chronic plaque psoriasis, another autoimmune disease mediated by the IL-17 pathway. Subsequently, similar positive efficacy for inhibition of IL-17A was seen in patients with ankylosing spondylitis and psoriatic arthritis. Inhibition of IL-17A may also improve cardiovascular and metabolic comorbidities often found in patients with SpA because studies have linked these disorders to the IL-17 pathway. In this review, we will examine key preclinical studies that demonstrated the mechanistic role of IL-17A in the development SpA and discuss how these observations were translated into clinical practice.

## Background

Spondyloarthritis (SpA) is a term used to describe a group of inflammatory autoimmune diseases that share common genetic risk factors and clinical features, including ankylosing spondylitis (AS), psoriatic arthritis (PsA), reactive arthritis, undifferentiated SpA, and enteropathic arthritis [1–6]. SpA typically affects the spine, sacroiliac joints, entheses, and peripheral joints. Hallmark features of SpA include enthesitis, focal inflammation, new bone formation and cartilage ossification that may lead to ankyloses [2, 4, 7–9]. Clinical phenotypes include inflammatory back pain, oligoarthritis, dactylitis, and extra-articular features such as uveitis, psoriasis, and inflammatory bowel disease (IBD) [5].

Worldwide estimates of the prevalence of SpA are variable. Available data suggest that SpA is relatively uncommon in Asian and Middle Eastern countries, affecting less than 0.9% of adult populations, while studies in European countries report SpA prevalence of between 0.3% and 1.9% [5]. According to data from the 2009–2010 US National Health and Nutrition Examination Survey (NHANES), it is estimated that SpA affects roughly 1% of the adult population in the US, which corresponds to between two million and three million people [6].

The exact pathogenesis of SpA is not well understood, but in recent years it has become clear that cytokines associated with innate and adaptive inflammatory immune responses play an important role in disease initiation and progression [2, 8, 9]. Cytokines produced by T-helper (Th)17 cells have been shown to contribute to pathogenic features of SpA, including joint erosion and inflammation, new bone formation, epidermal thickening, development of psoriatic skin lesions, pannus formation in joint capsules, synovial inflammation, and angiogenesis [7–12]. Specifically, preclinical studies using models of SpA have shown that expression of genes encoding proinflammatory cytokines including interleukin (IL)-17A, tumor necrosis factor (TNF)-α, and IL-23 is upregulated in affected joints [9, 13] and that IL-17 promotes osteogenesis at inflamed sites (e.g., entheses) under conditions of mechanical stress, resulting in excess bone formation that is a key characteristic difference between SpA and rheumatoid arthritis (RA), which is characterized predominantly by bone erosion and resorption [14]. As such, the IL-17 pathway has been identified as a potential disease-modifying target for the treatment of SpA [9, 12, 15]. This article provides a review of the pathogenesis of SpA and the mechanistic rationales for targeting the IL-17A pathway based on its role in inflammation, cartilage damage, and bone changes.

## Methods

Literature searches were conducted using PubMed to identify relevant articles and these results were supplemented by reference checking and with the authors’ familiarity of the literature. Due to the large number of articles discussing IL-17A in SpA, a narrative review was deemed to be most appropriate for this manuscript.

### IL-17A in the pathogenesis of SpA

**Fig. 1:**
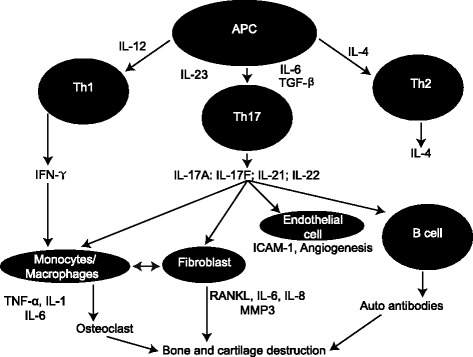
Pathogenic role of IL-17A in SpA [12]. Reprinted from Raychaudhuri SP. Role of IL-17 in psoriasis and psoriatic arthritis. Clin Rev Allergy Immunol. 2013;44:183–93, with permission of Springer. *APC* antigen-presenting cell, *ICAM-1* intercellular adhesion molecule-1, *IL* interleukin, *IFN* interferon, *MMP3* matrix metalloproteinase 3, *TGF* transforming growth factor, *RANKL* receptor activator of nuclear factor-κB ligand, *Th* T-helper, *TNF* tumor necrosis factor

IL-17 signaling has been identified as a key modulator of synovial inflammation and joint destruction in various arthropathies through its actions on synovial cells, osteoblasts, and chondrocytes (Fig. 1) [12]. Of the six members of the IL-17 cytokine family, IL-17A is the most well characterized, existing as a homodimer or as part of a heterodimer with IL-17F [8, 15]. Cellular production of IL-17 is complex and heterogeneous. Table 1 provides an overview of the diverse cellular sources of this cytokine. Notably, IL-17A and IL-17F are primarily produced by naive CD4^+^ T cells that differentiate into Th17 cells in response to stimulation by IL-23 [8, 16]. In addition, IL-17A is produced by CD8^+^ T cells, γδ T cells, natural killer cells, mast cells, neutrophils, and group 3 innate lymphoid cells [8]. Binding of IL-17A homodimers to the heterodimeric transmembrane IL-17 receptor (IL-17RA and IL-17RC) is a potent signal driving autoimmune inflammatory responses associated with arthritis-related tissue damage and joint destruction, as well as with mucocutaneous defense mechanisms against extracellular pathogens [8, 12, 14, 17, 18]. Furthermore, Th17 cells ( IL-17A/IL-22) and its receptor system play important roles in osteoclast-mediated bone changes and cartilage destruction associated with inflammatory arthritis [2, 9, 14]. The IL-17 receptor is expressed by osteoclasts, osteoblasts, synoviocytes, and chondrocytes, and stimulation of IL-17A triggers Th17 cells, osteoblasts, and synovial fibroblasts to produce the receptor activator of nuclear factor-κB ligand (RANKL), which plays a vital role in production of osteoclasts and osteoclastic precursors [14].

**Table 1:** Types of IL-17-producing cells [8, 12, 33, 36, 38, 76–80]

Cell type	Description
Adaptive Th17 cells	• A subset of activated CD4^+^ T helper cells that produce high levels of IL-17A, IL-17F, and IL-22, and express IL-23R • CD4^+^ TCRα/β^+^ Th17 cells are a well characterized source of IL-17 that play a key role in immune inflammatory responses
Natural Th17 cells	• Subset of thymic Th17 cells that acquire effector function prior to peripheral antigen exposure • These cells have different TCR gene usage and signaling properties compared with conventional Th17 cells
γ/δ T cells	• Potent source of innate IL-17 produced independently of IL-6 • Properties are similar to Th17 cells (e.g., expression of CCR6, IL-23R, and RORγt); these cells also express TLR1, TLR2, and Dectin-1 • Levels of IL-17-producing γ/δ T cells increase during some types of bacterial infections • Different subsets of γ/δ T cells in the thymus produce either IL-17 or IFNγ • Major source of gut-protective IL-17, which acts independently from IL-23
iNKT cells	• Cells that express a restricted TCR that recognizes glycolipid antigens • May provide an alternative source of IL-17 when IL-6 is not present to stimulate Th17 cells • IL-17^+^ cells express IL-23R and IL-1R1
Tc17 cells	• Subset of CD8^+^ cells that produces IL-17 • May play a role in pathogenic skin and joint inflammation in psoriasis and PsA, respectively
ILC3s	• Subset of ILCs defined by their capacity to produce IL-17A and/or IL-22 • The role of ILCs in SpA and other forms of destructive arthritis is unclear because cell numbers are generally low
Neutrophils	• Source of IL-17 in effector phase of arthritis • Myeloperoxidase^+^ and CD15^+^ neutrophils have been identified as sources of IL-17 in facet joints of patients with SpA
Mast cells	• Primary source of IL-17^+^ cells in synovial fluid of patients with SpA as a result of innate immune responses

*CCR6* C-C chemokine receptor type 6, *IFN* interferon, *IL* interleukin, *ILC* innate lymphoid cell, *iNKT cells* invariant natural killer T cells, *PsA* psoriatic arthritis, *ROR* retinoic orphan receptor, *SpA* spondyloarthritis, *TCR* T-cell receptor, *Th* T-helper, *TLR* Toll-like receptor

Many basic research studies using in vitro and animal models have helped elucidate the roles of IL-17A and related cytokines in arthritis pathogenesis. Early mouse models of collagen-induced arthritis (CIA) identified IL-17 as a key contributor to RANKL-induced bone erosion and inflammatory joint destruction [19, 20]. Subsequently, tissue microscopy studies have shown that T cells with high levels of IL-23 receptors are present at the entheseal interface between tendon and bone of both axial and peripheral articular locations [9]. When IL-23 levels were increased in cell cultures of these entheses, expression of IL-17A, IL-22, and bone morphogenic protein 7 increased, and IL-23 also induced severe enthesitis in animal models [9]. Initially, findings from genomic studies indicated that IL-23 receptor signaling plays a key role in the development of SpA, psoriasis, and IBD [21–24]. However, more recent studies in IL-17A^−/−^ knockout mice have clarified that IL-23-mediated stimulation of IL-17A and IL-22 drives the development and severity of enthesitis [21]. Further substantiating the role of IL-17A in IL-23-induced local inflammation, Reinhardt and colleagues showed that entheseal γ/δ cell production is increased under conditions of inflammation and mechanical stress, stimulating production of IL-17A at anatomic sites commonly affected in SpA (e.g., Achilles tendon insertion, aortic root, and ciliary body) [25].

Further supporting the role of IL-17A signaling in the formation of pathogenic bone erosions, joint inflammation, and cartilage destruction, Lubberts and colleagues [26] observed that, in a mouse model of CIA, neutralization of endogenous IL-17A was associated with a decrease in systemic IL-6 and a reduction in cells that tested positive for RANKL and the inflammatory cytokine IL-1β. In addition, Adamopoulos and colleagues [27] observed that IL-17A gene transfer in a mouse model of CIA induced the expansion of osteoclast precursors and increased serum levels of biomarkers associated with bone resorption, including tartrate-resistant acid phosphatase 5b and carboxy-terminal collagen crosslinks.

Findings consistent with the results from basic research studies have been observed in several different human studies in SpA. These studies have shown that levels of IL-17A, IL-23, IL-6, IL-1β, IL-21, transforming growth factor (TGF)-β, TNF-α, and interferon (IFN)γ are increased in sera and synovial fluid of patients with reactive arthritis, AS, and undifferentiated SpA, and that Th17 cell levels are increased in peripheral blood samples from patients with PsA and AS [3, 28–31]. Pathologic Th17-cell overexpression of IL-17 has also been observed in the gut of patients with PsA, contributing to chronic subclinical inflammation [32].

A study from our group that highlights the role of IL-17A signaling in inflammatory arthritis showed that elevated levels of IL-17A and IL-17RA are present in synovial tissue samples from patients with PsA and, when cultures of these tissue samples were treated with an anti-IL-17RA antibody, IL-17A-induced expression of IL-8, IL-6, and matrix metalloproteinase (MMP)3 was decreased [33]. Another important study by Jansen and colleagues [34] showed that elevated levels of IL-17-producing CD4^+^ T cells are present in patients with early, active axial SpA with and without magnetic resonance imaging (MRI) abnormalities. Furthermore, Kenna and colleagues [35] observed that patients with active AS have increased levels of circulating γδ T cells that express IL-23R and produce IL-17, and a study by Noordenbos and colleagues [36] showed that synovial tissue from patients with SpA has increased levels of IL-17-expressing mast cells compared with tissue from patients with RA. Interestingly, this increase in IL-17 was not modulated by TNF-α blockade [36]. These studies highlight just a few key examples of the extensive research findings that have established IL-17A signaling as an important pathway in the development and progression of SpA.

### Different roles of IL-17A in SpA and RA

While there are many similarities between the chronic spinal and joint inflammation observed in patients with RA and SpA, it is important to note that these diseases have several unique clinical, radiologic, serologic, and genetic features driven by different underlying pathogenic signaling pathways (Table 2) [8, 37]. Moreover, while many of the same cytokines are present in both RA and SpA, differential expression levels result in substantial differences in characteristic bone changes. In RA, TNF-α is the predominant proinflammatory cytokine, promoting RANKL-driven osteoclast erosion, as well as suppression of osteoblast formation [14]. In contrast, the IL-17A pathway plays a predominant role in the promotion of osteoclastogenesis in patients with SpA, and levels of circulating Th17 cells observed in the peripheral blood are much higher and more consistently observed than in patients with RA. Additionally, Th17 cells isolated from patients with SpA are more differentiated and polyfunctional than those from patients with RA [28]. These findings may be a result of different T-cell lineages in RA and SpA. For example, Menon and colleagues [38] observed that IL-17 in patients with PsA was predominantly produced by CD8^+^-derived T cells (Tc17 cells), while IL-17A-producing T cells were only produced from the CD4^+^ lineage in patients with RA. Furthermore, these authors observed that the frequency of synovial fluid IL-17^+^CD4^−^ T cells but not of IL-17^+^CD4^+^ T cells was associated with clinical measures of PsA disease activity [38].

**Table 2:** Differences between RA and SpA [8, 37]

RA	SpA
• Characterized primarily by symmetric polyarthritis and inflammation resulting in cartilage and bone destruction	• Oligo/polyarthritis is more often asymmetrical
• Axial involvement is rare	• Characterized primarily by axial disease of the sacroiliac joints and spine
• More common in women than men	• More common in men than women
• ACPA and RF antibodies are common	• ACPA and RF antibodies are absent
• Strong genetic association with HLA-DR	• Most common genetic involvement is HLA-B27
• Central clinical feature is synovitis	• Affects the axial skeleton and peripheral joints with synovial involvement, especially in entheses, bone, and bone marrow (osteitis)
• Driven by B- and/or T-cell autoreactivity	• Driven by innate immune cells (e.g., macrophages, PMN cells, mast cells)
• Macrophage effectors of synovial inflammation are driven predominantly by IFNγ	• Macrophage effectors of inflammation are driven predominantly by IL-17
• More pronounced hyperplasia of the synovial lining versus SpA	• Increased vascularity versus RA
• Extra-articular features include rheumatoid nodules, vasculitis, pneumonitis, scleritis	• Extra-articular features include IBD, psoriasis, uveitis, aortitis
• Little or no signs of tissue repair with joint damage	• Joint damage is characterized by new cartilage and bone formation (remodeling)

*ACPA* anti-citrullinated protein antibody, *HLA* human leukocyte antigen, *IBD* inflammatory bowel disease, *IFN* interferon, *IL* interleukin, *PMN* polymorphonuclear, *RA* rheumatoid arthritis, *RF* rheumatoid factor, *SpA* spondyloarthritis

### Translation of IL-17 inhibition into clinical practice

There is currently an unmet need in the treatment of SpA [39]. Traditional disease-modifying antirheumatic drugs (e.g., methotrexate) are ineffective in spinal inflammatory arthritis and AS. While the TNF-α inhibitors etanercept, adalimumab, infliximab, certolizumab pegol, and golimumab have all demonstrated efficacy in AS and PsA, a substantial proportion of patients either do not respond at all or respond inadequately, while some patients are unable to tolerate these drugs [15, 39–41]. In recent years, other therapies used to treat RA have been investigated in AS, including the IL-1 receptor antagonist anakinra, the IL-6 inhibitor tocilizumab, the anti-CD20 antibody rituximab, and the costimulatory pathway inhibitor abatacept. However, none of these agents have demonstrated a meaningful clinical benefit in patients with AS [41].

It has been recognized that biologic agents with mechanisms of action other than TNF inhibition, which acts upstream of newer agents, would provide valuable alternatives for the treatment of SpA. However, until very recently there was a lack of alternatives to TNF-α inhibitors [39]. That changed with the approval of the IL-12/IL-23 antagonist ustekinumab, the phosphodiesterase 4 inhibitor apremilast, and the IL-17A inhibitor secukinumab for the treatment of PsA, and with the approval of secukinumab for the treatment of AS. Now, when patients fail to respond to one biologic due to lack of efficacy or poor tolerability, switching to another biologic with a different mechanism of action can be an effective treatment strategy [42, 43].

When choosing a biologic agent in SpA, it is reasonable to consider preclinical findings that support the mechanistic rationale for targeting IL-17A. IL-17A seems to be a more downstream effector of SpA pathogenesis than other cytokines (i.e., IL-23, TGF-β, IL-6, and IL-1β), which act upstream to trigger differentiation of Th17 cells in entheses and synovial tissue. Once activated, these Th17 cells stimulate production of downstream effector cytokines including IL-17 and IL-22, which contribute to inflammation, bone erosion, and bone fusion [2, 7, 9, 12, 27, 28, 33]. In addition to the good efficacy observed in SpA with this strategy, such downstream targeting may also be beneficial for patients with refractory SpA and those who are otherwise intolerant to other biologic therapies.

As clinical development programs for IL-17A inhibitors have progressed, findings from large-scale randomized controlled trials have supported the translation of IL-17A inhibition in SpA from bench to bedside. Positive findings have been observed in clinical studies of IL-17A inhibitors in psoriasis, PsA, and AS [44–49].

#### Psoriasis

The IL-17A inhibitor secukinumab is approved for the treatment of moderate-to-severe plaque psoriasis. In phase 3 clinical studies, secukinumab consistently provided substantial meaningful improvements in the signs and symptoms of moderate-to-severe chronic plaque psoriasis [44, 49]. In the pivotal ERASURE and FIXTURE studies, more than half of patients who received secukinumab 300 mg experienced a 90% improvement in Psoriasis Area and Severity Index (PASI90) scores at week 12, compared with 1% with placebo (ERASURE) and 21% with etanercept (FIXTURE) [44]. In the CLEAR phase 3 study, secukinumab 300 mg demonstrated superiority compared with ustekinumab for the primary endpoint of PASI90 at week 16 (79.0% vs 57.6% response rates, respectively; *p* < 0.0001) [49].

The IL-17A inhibitor ixekizumab was recently approved for the treatment of moderate-to-severe plaque psoriasis. In the UNCOVER-2 and UNCOVER-3 randomized controlled phase 3 studies, up to 71% of patients treated with ixekizumab achieved PASI90 at week 12, compared with 0.6% to 3.1% with placebo, and 18.7% to 25.7% with etanercept [45].

The anti-IL-17RA monoclonal antibody brodalumab was in late-stage development for the treatment of moderate-to-severe plaque psoriasis. Results of the phase 3 AMAGINE-1 study showed that, at week 12, PASI90 was achieved by 43% of patients treated with brodalumab 140 mg and by 70% of patients treated with brodalumab 210 mg, compared with 1% of patients in the placebo group [50]. In the AMAGINE-2 and AMAGINE-3 studies, brodalumab 210 mg demonstrated higher efficacy compared with ustekinumab based on week 12 PASI90 responses (70% vs 47% in AMAGINE-2, and 69% vs 48% in AMAGINE-3) [51]. However, the future of brodalumab development is uncertain based on observed events of suicidal ideation and behavior during clinical development [52].

#### Psoriatic arthritis

In early 2016, secukinumab received US Food and Drug Administration (FDA) approval for the treatment of active PsA. In the large-scale FUTURE 1 and FUTURE 2 phase 3 studies, patients received placebo or intravenous (IV) or subcutaneous (SC) induction dosing of secukinumab followed by SC maintenance dosing [46, 48]. At week 24 in FUTURE 1, at least 20% improvement in American College of Rheumatology response criteria (ACR20) responses were observed in 50% of patients receiving secukinumab 150 mg, 51% receiving secukinumab 75 mg, and 17% of patients receiving placebo (*p* < 0.001 for both comparisons with placebo) [48]. At week 24 in FUTURE 2, which had a mixed population of TNF-naive and TNF-experienced patients, ACR20 was achieved by 54% of patients receiving secukinumab 300 mg (*p* < 0.0001), 51% receiving secukinumab 150 mg (*p* < 0.0001), 29% receiving secukinumab 75 mg (*p* = 0.0399), and 15% of patients receiving placebo [46]. These improvements were sustained through 52 weeks of treatment [46, 48].

In the phase 3 SPIRIT-P1 trial of a TNF-naive population, significantly more patients achieved ACR20 with ixekizumab 80 mg once every 2 weeks (62%) and ixekizumab 80 mg once every 4 weeks (58%) than with placebo (30%; both *p* ≤ 0.001) [53]. A clinical trial of ixekizumab in biologic-experienced patients with active PsA is ongoing (NCT02349295).

#### Ankylosing spondylitis

Secukinumab also recently received FDA approval for the treatment of active AS. In a randomized, double-blind, phase 2 study, 59% of patients treated with secukinumab achieved rapid, meaningful clinical improvement in AS symptoms compared with 24% of patients who received placebo [54]. These improvements were maintained for up to 2 years in patients who continued in an open-label extension of this study [55]. The benefits of secukinumab in patients with AS were confirmed in the phase 3 MEASURE 1 and MEASURE 2 studies [47]. In these randomized controlled trials, patients received placebo, or IV or SC loading doses of secukinumab followed by SC maintenance dosing. In MEASURE 1 and MEASURE 2, respectively, at least 20% improvement in Assessment of SpondyloArthritis International Society (ASAS20) response rates at week 16 were 61% in both studies in the secukinumab 150 mg group, 60% and 41% in the secukinumab 75 mg group, and 29% and 28% for patients who received placebo [47]. Improvement in signs and symptoms of AS occurred rapidly with secukinumab and were sustained over time. Secukinumab was also associated with improvements in physical functioning and health-related quality of life compared with placebo [47].

Studies of ixekizumab in biologic treatment-naive (COAST-V) and treatment-experienced (COAST-W) patients with AS are ongoing (NCT02696785 and NCT02696798).

#### Rheumatoid arthritis

As suggested by basic research studies characterizing the different roles of IL-17A in RA and SpA [38], results from clinical studies of IL-17A inhibitors in RA indicate that these agents may have limited therapeutic efficacy in this patient population [56, 57]. In separate phase 2 studies of secukinumab and ixekizumab in patients with RA, ACR20 response rates differed across the dose ranges studied, without a consistent dose–response relationship [56, 57]. In a 16-week phase 2 study of secukinumab, ACR20 response rates ranged from 36% to 54% in the active-treatment arms, compared with 34% with placebo [56]. In a second 52-week phase 2 study of secukinumab, ACR20 response rates were not significantly different between secukinumab and placebo (42.9% vs 40.9%), but significant improvements were observed in the secukinumab arm based on 28-joint Disease Activity Score (DAS28), global assessments of disease activity, pain assessments, and high-sensitivity C-reactive protein levels [58].

Among biologic treatment-naive patients in a phase 2 study of ixekizumab, ACR20 response rates ranged from 43% to 70%, compared with 35% for placebo. In this study, ACR20 response rates were significantly higher with ixekizumab 80 mg and 180 mg compared with placebo for patients with prior inadequate response to TNF-α inhibitors [57]. Patients in this study were eligible to enter a 48-week open-label extension. Patients in the extension who experienced clinical improvements during the initial 16-week study generally maintained these improvements through week 64 [59].

Based on findings from these phase 2 studies, it cannot be ruled out that a subset of patients with RA may experience clinical benefits from treatment with IL-17A inhibitors. However, a recent study investigating the relationship between genetic biomarkers and secukinumab treatment response in patients with RA found that the human leukocyte antigen (HLA)-DRB1*04 and HLA-DRB1* shared epitope allelic groups were not predictive of treatment response [60].

#### Safety profiles of secukinumab and ixekizumab

IL-17A inhibitors have been shown to have good overall safety profiles [61]. In psoriasis clinical trials, secukinumab’s side-effect profile was similar to that of the TNF-α inhibitor etanercept and the IL-12/IL-23 antagonist ustekinumab, with no safety signals indicating an increased risk for serious adverse events of interest, including malignancy, infection, or induction of autoimmune diseases [44, 49]. In placebo-controlled studies of secukinumab in PsA and AS, the incidence and types of adverse events were similar between secukinumab and placebo, with a small increased risk for infections, including candidiasis [46–48]. Similarly, in the UNCOVER-2 and UNCOVER-3 psoriasis clinical studies, rates of serious adverse events were low and similar with ixekizumab and etanercept, and a small number of *Candida* infections were reported [45].

### Management of comorbidities and extra-articular manifestations of SpA

It has been hypothesized, based on findings from recent research, that IL-17A inhibition may have beneficial effects on some of the common comorbidities observed in patients with SpA, as well as extra-articular symptoms of the disease, including uveitis and psoriasis [62]. Based on available data, Golden and colleagues [63] have speculated that the inflammation associated with increased levels of Th17 cells and their associated cytokines may initiate or worsen comorbidities, including atherosclerosis and metabolic syndrome; thus, it is logical to hypothesize that SpA treatments that modulate inflammatory responses in the Th17 pathway could have added benefits related to improvements in comorbidities.

In experiments performed by Barry and colleagues, prolonged elevations in IL-17RA, IL-17A, and IL-17F levels following myocardial ischemia/reperfusion injury in rats contributed to permanent cardiac damage [64]. In vivo experiments also showed that IL-17 neutralization reduced necrotic and apoptotic myocyte death, suggesting beneficial effects following human myocardial infarction [64].

Preclinical studies have linked increased IL-17 production with development of hypertension and endothelial dysfunction through promotion of oxidative stress in the kidneys and vasculature [65, 66]. These findings are supported by results from a clinical study that showed serum IL-17 levels were significantly higher in patients with prehypertension compared with normotensive controls [67]. Additionally, Hautefort and colleagues [68] observed that monocyte-derived dendritic cells in patients with idiopathic pulmonary arterial hypertension induced greater activation and proliferation of CD4^+^-derived Th17 cells, resulting in higher levels of IL-17 compared with controls.

In a large-scale, case-controlled, genetic screening study in patients with coronary artery disease (CAD), Zhang and colleagues [69] found a significant association between specific *IL17A* single nucleotide polymorphisms (SNPs) and risk of CAD and acute myocardial infarction in a Chinese Han population. Similarly, Vargas-Alarcón and colleagues [70] found associations between two *IL17A* haplotypes and development of premature CAD in a Mexican population. In the group that developed premature CAD, polymorphisms were also associated with increased risks for central obesity, increased visceral abdominal fat, and metabolic syndrome. Interestingly, such increased risks for obesity may contribute to disease pathogenesis in SpA because activated white adipose tissue stimulates production of IL-17 and other proinflammatory cytokines [71].

In a randomized, placebo-controlled, phase 2a trial of patients with moderate-to-severe Crohn’s disease, IL-17A inhibition with secukinumab did not meet its primary endpoint, and worsening of Crohn’s disease was experienced by some patients [72]. However, in mechanistic studies, mixed findings have been reported for the role of IL-17 in the gut with both protective and proinflammatory effects observed [73] and additional research is required to fully understand the role of IL-17 in the gut. Although Crohn’s disease has been reported in patients with AS treated with IL-17 inhibitors, a small number of individuals were affected (incidence rate of 0.7 cases per 100 patient-years in pooled secukinumab-group data from MEASURE 1 and MEASURE 2) [47]. Furthermore, in large, pooled studies of patients with psoriasis treated with IL-17 inhibitors, IBD was reported infrequently (incidence rate of 0.33 cases per 100 patient-years with secukinumab and incidence rate of 2.9 cases per 1000 patient-years with ixekizumab) [74, 75]. The risk of IBD with IL-17 inhibition is minimal.

## Conclusions

In summary, there is ample evidence supporting the central role of IL-17A-secreting cells in inflammation, bone resorption/remodeling, and cartilage damage associated with SpA, and there are strong mechanistic rationales for targeting this pathway in SpA disease management. Findings from clinical studies support these rationales and indicate that biologic IL-17A inhibitors may provide effective and well-tolerated alternatives to other approved treatments for the management of SpA.

## Abbreviations

ACR: American College of Rheumatology
AS: Ankylosing spondylitis
ASAS: Assessment of SpondyloArthritis International Society
CAD: Coronary artery disease
CIA: Collagen-induced arthritis
DAS28: 28-joint Disease Activity Score
FDA: Food and Drug Administration
HLA: Human leukocyte antigen
IBD: Inflammatory bowel disease
IFN: Interferon
IL: Interleukin
IL-17RA: Interleukin-17 receptor A
IL-17RC: Interleukin-17 receptor C
IV: Intravenous
MMP: Matrix metalloproteinase
NHANES: National Health and Nutrition Examination Survey
PASI: Psoriasis Area and Severity Index
PsA: Psoriatic arthritis
RA: Rheumatoid arthritis
RANKL: Receptor activator of nuclear factor-κB ligand
SC: Subcutaneous
SNP: Single nucleotide polymorphism
SpA: Spondyloarthritis
TGF: Transforming growth factor
Th: T-helper
TNF: Tumor necrosis factor
